# Multifactorial Benefits of Chronic Vagus Nerve Stimulation on Autonomic Function and Cardiac Electrical Stability in Heart Failure Patients With Reduced Ejection Fraction

**DOI:** 10.3389/fphys.2022.855756

**Published:** 2022-03-30

**Authors:** Richard L. Verrier, Imad Libbus, Bruce D. Nearing, Bruce H. KenKnight

**Affiliations:** ^1^Beth Israel Deaconess Medical Center, Department of Medicine, Division of Cardiovascular Medicine, Harvard Medical School, Boston, MA, United States; ^2^LivaNova USA, Inc., Houston, TX, United States

**Keywords:** heart failure, vagus nerve stimulation, autonomic regulation therapy, heart rate variability, heart rate turbulence, T-wave alternans, R-wave heterogeneity, T-wave heterogeneity

## Abstract

Heart failure with reduced left ventricular ejection fraction is a progressive disease that claims > 352,000 lives annually in the United States alone. Despite the development of an extensive array of pharmacologic and device therapies, prognosis remains poor. Disruption in autonomic balance in the form of heightened sympathetic nerve activity and reduced vagal tone have been established as major causes of heart failure progression. Interest in chronic neuromodulation mediated by vagus nerve stimulation (VNS) has intensified in recent years. This review focuses on four main goals: (1) To review the preclinical evidence that supports the concept of a cardioprotective effect of VNS on autonomic function and cardiac electrical stability along with the underlying putative mechanisms. (2) To present the initial clinical experience with chronic VNS in patients with heart failure and highlight the controversial aspects of the findings. (3) To discuss the latest findings of the multifactorial effects of VNS on autonomic tone, baroreceptor sensitivity, and cardiac electrical stability and the state-of-the-art methods employed to monitor these relationships. (4) To discuss the implications of the current findings and the gaps in knowledge that require attention in future investigations.

## Introduction

Heart failure with reduced ejection fraction (HFrEF) is a progressive disease associated with increased morbidity and mortality. HFrEF is a major cause of death in the United States, resulting in an estimated > 352,000 deaths annually (Virani et al., 2020). A diverse armamentarium of pharmacologic and device therapies have been developed with notable success. Unfortunately, prognosis remains poor as it has been challenging to curtail long-term deterioration of autonomic and cardiac electrical function. Because disruptions in autonomic balance due to heightened sympathetic nerve activity and reduced parasympathetic activity have been implicated in increased morbidity and mortality in heart failure (Zhang and Anderson, 2014), there has been a strong interest in developing chronic methods for neuromodulation, both with drugs and devices, to mitigate major pathophysiological factors.

Vagus nerve stimulation (VNS) has been the focus of intensive study because of its potential for cardioprotective effects (De Ferrari and Schwartz, 2011). An attractive feature of VNS is its established safety profile and tolerability, documented in the treatment of > 120,000 individuals with epilepsy or depression over more than three decades (Conway et al., 2015). The salutary effects of chronic VNS in drug-resistant epilepsy have been found to persist for > 10 years (Ryvlin et al., 2018). VNS is also capable of reducing cardiac electrical instability as demonstrated by its capacity to suppress T-wave alternans (TWA), a marker of cardiac instability, in patients with pharmacoresistant epilepsy (Schomer et al., 2014; Verrier et al., 2016, 2020). This observation was somewhat surprising as in epilepsy patients, VNS electrodes are oriented in a cephalad configuration (negative electrode toward the head and positive electrode toward the chest) to optimize central nervous system effects for seizure reduction. The caudad orientation of the electrodes, placed primarily on the right vagus nerve, in contrast to the left vagus nerve in epilepsy, has been employed in patients with heart failure. There are also differences in stimulation parameters in these two clinical applications. In the future, a challenge will be to optimize stimulation methodology in epilepsy patients to achieve both anti-seizure effects and cardioprotection.

### Main Objectives of the Review

The main goals are four-fold: (1) To review the preclinical evidence that supports the concept of a cardioprotective effect of VNS on autonomic function and cardiac electrical stability along with the underlying putative mechanisms. (2) To present the initial clinical experience with chronic VNS in patients with heart failure and highlight the controversial aspects of the findings. (3) To discuss the latest findings of the multifactorial effects of VNS on autonomic tone, baroreceptor sensitivity, and cardiac electrical stability and the state-of-the-art methods employed to monitor these relationships. (4) To discuss the implications of the current findings and the gaps in knowledge that require attention in future investigations.

## Experimental Studies of the Effects of Vagus Nerve Stimulation on Autonomic Tone and Vulnerability to Malignant Cardiac Arrhythmias

More than a century and a half ago, Einbrodt (1859) utilized an inductorium to assess the effects of vagus nerve excitation on the canine heart. During VNS, the electrical current intensity required to induce ventricular fibrillation was elevated. The capacity of concomitant VNS to suppress ventricular fibrillation remained relatively underappreciated, as textbook information suggested that vagal influences were limited to supraventricular structures. However, Kent et al. (1974) provided definitive evidence based on anatomic and electrophysiologic measures that there is rich cholinergic innervation of the atrioventricular node and ventricular conducting system not only in canines but also in humans. This observation carried important implications and stimulated a number of experimental studies confirming the initial insight provided by Einbrodt’s pioneering observation that VNS could protect against ventricular fibrillation induction.

**FIGURE 1:**
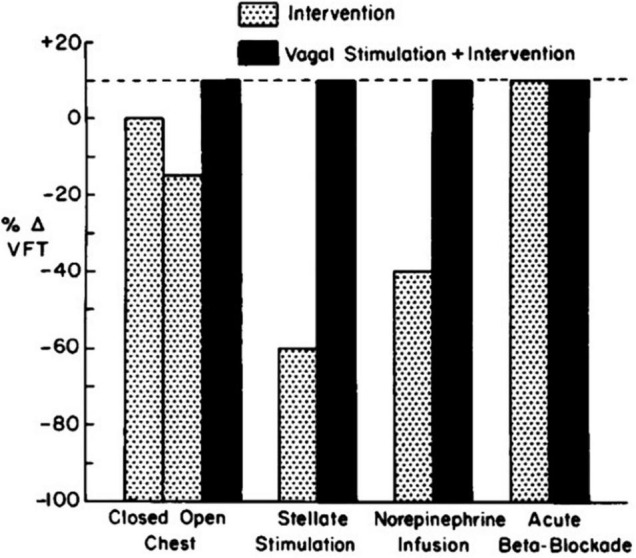
Influence of vagal stimulation in the presence of various levels of adrenergic tone. The vagal effect on ventricular fibrillation threshold (VFT) is demonstrable only when neural or humoral activity is increased (Lown and Verrier, 1976).

Over the course of more than a decade, researchers provided additional evidence of the multiple modes of action whereby VNS helps to prevent ventricular fibrillation. Among the most fundamental discoveries is that vagal influences are contingent on the prevailing level of adrenergic tone (Kolman et al., 1975; Lown and Verrier, 1976; Matta et al., 1976; Rabinowitz et al., 1976; Danilo et al., 1978; Figure 1). Specifically, it was shown that when cardiac-bound sympathetic activity is increased by thoracotomy (Kolman et al., 1975), sympathetic nerve stimulation (Kolman et al., 1975), myocardial ischemia, or catecholamine infusion (Rabinowitz et al., 1976), VNS exerts an antifibrillatory effect, but is devoid of such an action when adrenergic input to the heart is suppressed by beta-adrenergic blockade (Lown and Verrier, 1976). Levy and Blattberg (1976) coined this concept “accentuated antagonism.” The mechanistic basis for accentuated antagonism of adrenergic effects is presynaptic inhibition of norepinephrine release from nerve endings and muscarinically mediated effects at the second messenger level, which in turn attenuate the response to catecholamines. In addition, vagal influences provide indirect protection against ventricular fibrillation by blunting excessive heart rates (Lown and Verrier, 1976), which can decrease diastolic perfusion time during acute myocardial ischemia.

It is important to note that the beneficial effects of vagus nerve activity may be annulled if profound bradycardia and hypotension are induced *via* high-frequency current stimulation. Vanoli et al. (1991) demonstrated a potent antifibrillatory effect of VNS during exercise-induced myocardial ischemia in canines with a healed myocardial infarction. Stimulation of the right cervical vagus nerve *via* chronically implanted electrodes following the onset of exercise-induced acute myocardial ischemia decreased the incidence of ventricular fibrillation by 92%. This effect was only partly due to the heart rate reduction that occurred concurrently with the antifibrillatory effect of VNS. In half of the animals, the protective effect of VNS persisted despite fixation of heart rate by atrial pacing, indicating some rate-independent antifibrillatory action.

**FIGURE 2:**
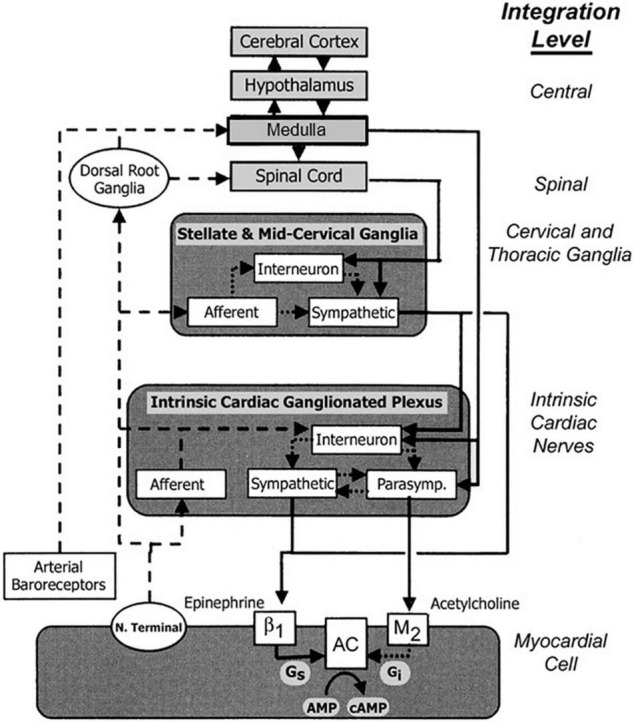
Traditional concepts focused on afferent tracts (*dashed lines*) arising from myocardial nerve terminals and reflex receptors (e.g., baroreceptors) that are integrated centrally within hypothalamic and medullary cardiostimulatory and cardioinhibitory brain centers and on central modulation of sympathetic and parasympathetic outflow (*solid lines*) with little intermediary processing at the level of the spinal cord and within cervical and thoracic ganglia. More recent views incorporate additional levels of intricate processing within the extraspinal cervical and thoracic ganglia and within the cardiac ganglionic plexus, where interneurons are envisioned to provide levels of non-central integration. Release of neurotransmitters from postganglionic sympathetic neurons is believed to enhance excitation in the sinoatrial node and myocardial cells through norepinephrine binding to β_1_ receptors, which enhances adenyl cyclase (AC) activity through intermediary stimulatory G proteins (Gs). Increased parasympathetic outflow enhances postganglionic release and binding of acetylcholine to muscarinic (M2) receptors, and through coupled inhibitory G proteins (Gi), inhibits cAMP production. The latter alters electrogenesis and pacemaking activity by affecting the activity of specific membrane Na, K, and Ca channels. New levels of integration are shown superimposed on previous views and are emphasized here to highlight new possibilities for intervention. Reproduced with permission from Wiley from Lathrop and Spooner (2001).

**FIGURE 3:**
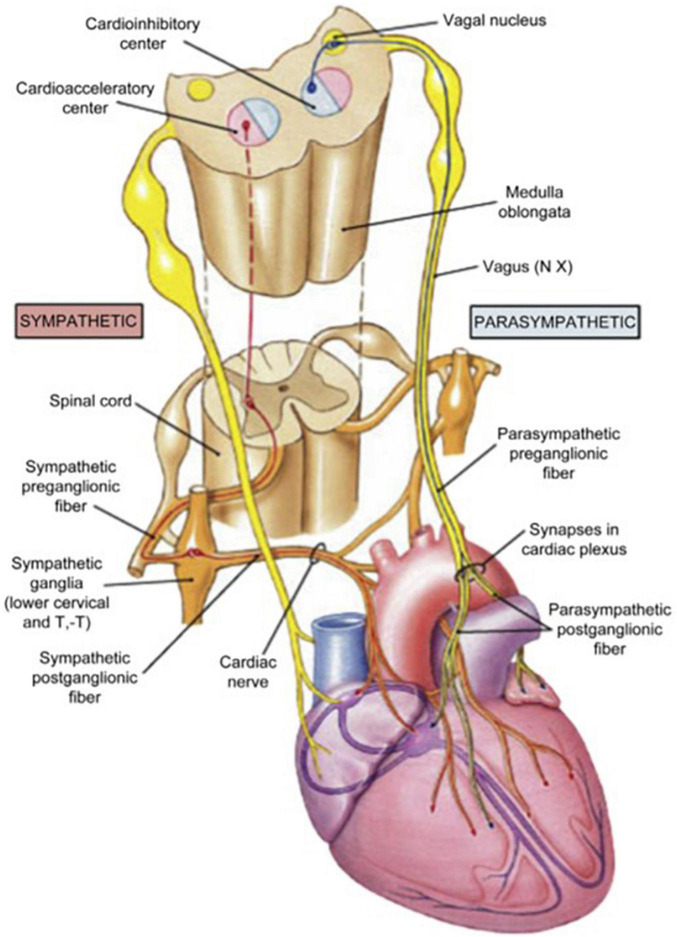
Anatomic organization of the extra-cardiac limbs of the autonomic nervous system (ANS)—the cardio-acceleratory sympathetic and cardio-inhibitory parasympathetic systems. Reprinted with permission from Pearson Education, Inc., from Martini and Nath (2009).

Schwartz, De Ferrari, and others (Schwartz et al., 2008; De Ferrari and Schwartz, 2011; De Ferrari et al., 2011) reviewed additional effects that underlie the multifactorial protective actions of VNS potentially relevant to treatment of heart failure. Specifically, although circulating cytokine levels are typically increased in patients with HFrEF (Deswal et al., 2011), VNS decreased circulating cytokine levels in an experimental heart failure model (Sabbah et al., 2011). VNS improved heart rate variability (HRV), increased baroreflex sensitivity (Zhang et al., 2009; De Ferrari et al., 2011), attenuated systemic inflammation (Pavlov et al., 2003), improved coronary flow (Henning and Sawmiller, 2001), and was antiapoptotic (Uemura et al., 2007; Beaumont et al., 2015). Vagal inhibition of the inflammatory reflex was shown to have the potential to suppress macrophage activation and synthesis of tumor necrosis factor (Yancy et al., 2017). The cardioprotective effects of VNS elucidated by experimental studies are summarized in Table 1. The multi-tiered loci within the neuraxis at which cervical vagal stimulation can act are illustrated in Figures 2, 3 (Lathrop and Spooner, 2001; Martini and Nath, 2009).

**TABLE 1:** Multifactorial cardioprotective effects of chronic low-level vagus nerve stimulation relevant to heart failure.

• Anti-inflammatory actions
• Augmentation of nitric oxide expression
• Reduction in apoptosis
• Inhibition of norepinephrine release at the cardiac neuroeffector junction
• Suppression of stellate ganglion nerve activity
• Improvement in autonomic balance and baroreceptor sensitivity, reduction in T-wave alternans and heterogeneity
• Corresponding improvement in LVEF, NYHA Class, suppression of ventricular arrhythmia

## Clinical Studies of the Effects of Chronic Vagus Nerve Stimulation in Patients With Heart Failure With Reduced Ejection Fraction

Schwartz et al. (2008) took the critical step of performing the first-in-human proof-of-concept study in eight patients with heart failure. The investigation demonstrated that VNS improved heart failure symptoms and reduced left ventricular end systolic volumes (LVESV). The patients experienced relatively minor adverse effects, including cough and dysphonia. Any stimulation-related sensations resolved over time. A subsequent phase II trial, the CardioFit Study (BioControl Medical Ltd., Yehud, Israel) (De Ferrari et al., 2011), enrolled 32 patients with heart failure in whom a VNS system using a right ventricular endocardial sensing electrode had been implanted to permit synchronization of VNS with the cardiac cycle to maximize vagal effects on the cardiovascular system. Overall, the investigation revealed an improvement in left ventricular ejection fraction (LVEF) from 22 to 29% (*p* < 0.001) and a reduction in LVESV at 6 and 12 months of treatment. These encouraging results led to the INcrease Of VAgal TonE in Heart Failure (INOVATE-HF) Study, which also used the CardioFit System and was a multinational, randomized controlled study enrolling 707 subjects with chronic heart failure and New York Heart Association (NYHA) Class III symptoms and LVEF < 40% (Gold et al., 2016). The patients were randomized to high-amplitude, low-frequency (1–2 Hz) VNS plus guideline-directed medical therapy vs. guideline-directed medical therapy alone. INOVATE-HF was halted because of futility in meeting the primary endpoint of reduction in all-cause mortality or first heart failure events despite significant improvements in secondary endpoints, namely, NYHA class, quality of life, and 6-min walk test. *Post hoc* analysis of the INOVATE-HF study revealed that adequate stimulation levels were not attained in all patients and that an adequate response to VNS was observed in only ∼30% of subjects (Anand et al., 2020).

The Neural Cardiac Therapy for Heart Failure Study (NECTAR-HF) (Boston Scientific, Marlborough, MA, United States) was a multi-center, randomized, sham-controlled study of low-amplitude, high-frequency VNS in 96 patients with chronic heart failure, NYHA Class II or III, and LVEF ≤ 35% (Zannad et al., 2015). It showed significant improvements in health-related quality of life and NYHA class after 6 months. VNS did not improve left ventricular end-systolic dimension, the primary efficacy endpoint, or other echocardiographic measures. The fact that VNS was delivered at a high frequency (20 Hz) limited the option to raise current amplitude due to adverse effects, i.e., cough, voice alteration, and discomfort. A subsequent analysis indicated that in the majority of patients, no significant increase was observed in HRV, a marker of physiological autonomic modulation (De Ferrari et al., 2017).

**FIGURE 4:**
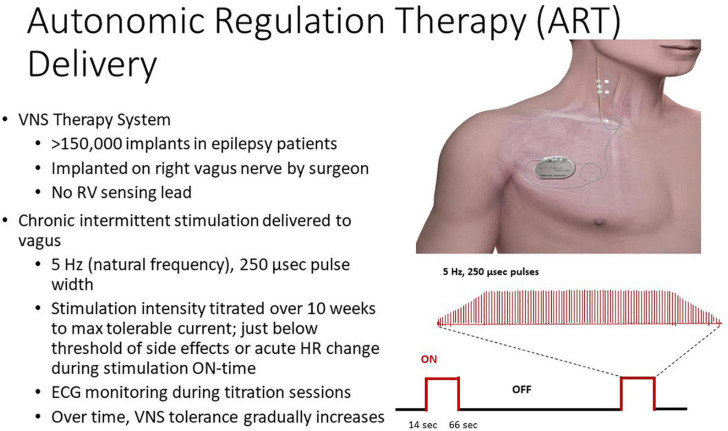
Autonomic regulation therapy (ART) system and approach. Reproduced with permission from LivaNova USA.

The Autonomic Regulation Therapy to Enhance Myocardial Function and Reduce Progression of Heart Failure (ANTHEM-HF) Pilot study (Houston, TX, United States), which enrolled 60 patients, demonstrated the safety and tolerability of VNS, reporting significant longitudinal improvements, including left ventricular function, 6-min walk test, patient-reported outcomes, and HRV (Premchand et al., 2014, 2016; Sharma et al., 2021). This study group employed “autonomic regulation therapy” to activate the vagus nerve within the optimum zone of the neural fulcrum (Ardell et al., 2017), a heart rate response zone where the central and peripheral functional effects of VNS are balanced. The basic approach employed in ANTHEM-HF is illustrated in Figure 4. The titration protocol employed moderate stimulation intensity (2.0 ± 0.6 mA) and frequency (5–10 Hz), which is near the “natural frequency” of discharge of cardiac vagal fibers during physiological reflex activation (Jewett, 1964). As a result, autonomic engagement with minimum heart rate alteration occurred during the “on” phase of VNS (Ardell et al., 2017). The VNS settings employed in ANTHEM-HF as compared to CardioFit, INOVATE-HF and NECTAR-HF are shown in Table 2. It is noteworthy that the latter three studies used VNS parameters that were well outside this neurophysiologically based therapeutic range.

**TABLE 2:** Comparative VNS settings in different heart failure studies.

	Implant Side	Pulse width (μsec)	Frequency (Hz)	Mean post-titration intensity (mA)	Duty Cycle [% (on/on + off) time]
ANTHEM-HF Pilot	Right or left	250	10 and 5	2.0 ± 0.6	18%
INOVATE-HF	Right	500–1000	1.5–2.0	3.9	18%
NECTAR-HF	Right	300	20	1.2	17%

## Multiyear Improvement in Autonomic Tone and Reflexes by Vagus Nerve Stimulation in the Autonomic Regulation Therapy to Enhance Myocardial Function and Reduce Progression of Heart Failure Pilot Study

Measures of autonomic function were assessed from 24-h AECGs recorded from 25 patients enrolled in the ANTHEM-HF Pilot study (Libbus et al., 2016; Nearing et al., 2021a). Significant increases to normal ranges were observed by 12 months in two HRV measures, namely, square root of the mean of the squares of the successive differences between adjacent beats (rMSSD) and high-frequency HRV.

**FIGURE 5:**
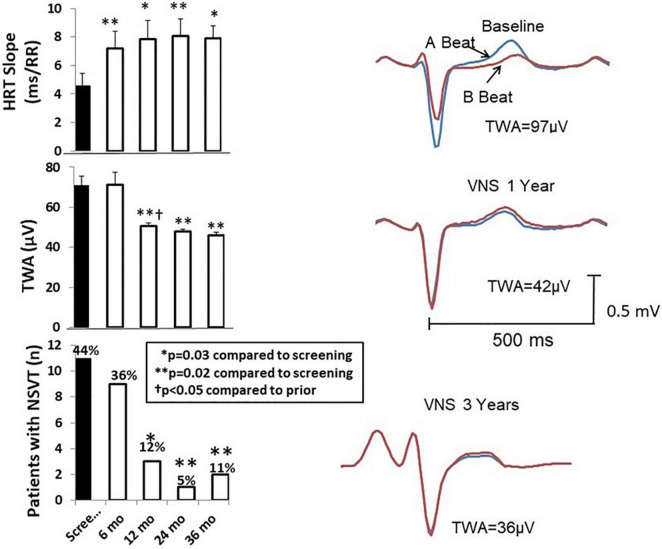
**Left panel:** Time course of effects of vagus nerve stimulation (VNS) therapy on heart rate turbulence (HRT) slope **(upper panel)**, T-wave alternans (TWA) **(middle panel)**, and numbers of patients with non-sustained ventricular tachycardia (NSVT) > 3 beats **(lower panel)**. The increase in HRT slope is associated with the beneficial effect of increasing baroreceptor sensitivity. Mean TWA was reduced from > 70 μV, a severely abnormal level (≥60 μV), to a normal range (<47 μV). The numbers of patients with NSVT were also significantly decreased by chronic VNS therapy. **Right panel:** TWA recordings from a representative patient at baseline screening and 1 and 3 years after VNS device implantation. Reproduced with permission from Elsevier from Nearing et al. (2021a).

A substantial and lasting increase in heart rate turbulence (HRT) slope, an indicator of baroreceptor sensitivity that is associated with reduced cardiovascular mortality (Bauer et al., 2008), was also noted as early as 6 months and was maintained throughout the 3 years of study (Figure 5, Nearing et al., 2021a). Another index of baroreceptor sensitivity, namely, intrinsic heart rate recovery, was also improved in the 3-year analysis (Nearing et al., 2021b).

The VNS-mediated increases in rMSSD and high-frequency HRV are likely to reflect an increase in cardiac-bound vagus nerve tone, resulting from excitation of vagal efferent fibers. However, the mechanisms whereby VNS improves HRT slope and intrinsic heart rate recovery are less certain. A plausible explanation is that the presumed enhancement in baroreceptor activity results in activation of vagal afferent fibers, which in turn influence the activity of the dorsal motor nucleus of the vagus nerve. The evidence supporting this possibility is illustrated in Figure 3 (Martini and Nath, 2009) and discussed in a review by Chatterjee and Singh (2015). The fact that VNS is effective in suppressing epileptic seizures (Ryvlin et al., 2018) supports the potent action of afferent vagal activity on the central nervous system.

## Sustained Beneficial Effects of Vagus Nerve Stimulation on T-Wave Alternans, R-Wave and T-Wave Heterogeneity, and Non-Sustained Ventricular Tachycardia

**FIGURE 6:**
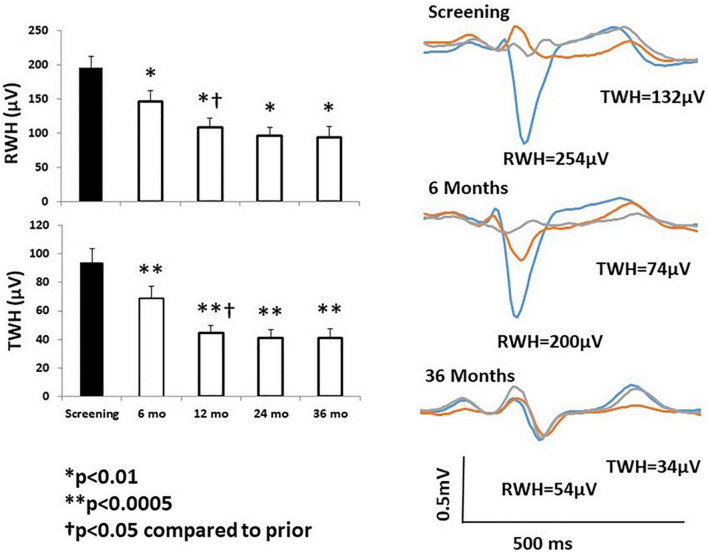
**Left panels:** Significant improvement in R-wave and T-wave heterogeneity (RWH, TWH) occurred at 6 months following initiation of chronic vagus nerve stimulation (VNS) and persisted throughout the study. **Right panel:** Substantial, sustained reductions RWH and TWH in a representative study patient. Tracings were obtained at baseline screening and at 6 and 36 months after VNS device implantation. Reproduced with permission from Elsevier from Nearing et al. (2021a).

Chronic VNS produced long-lasting beneficial effects on several markers of susceptibility to malignant cardiac arrhythmias during the three-year ANTHEM-HF Pilot study (Libbus et al., 2016; Nearing et al., 2021a). Specifically, TWA levels at baseline averaged 71 ± 4.6 μV in the 25 patients studied (Figure 5, middle left panel; Nearing et al., 2021a). This TWA level is in the severely abnormal range (≥60 μV), a magnitude associated with heightened risk for cardiac mortality and sudden cardiac death (Verrier et al., 2011; Verrier and Ikeda, 2013). At 12 months, a significant 20-μV (29%) decrease in TWA to nearly normal levels was observed (Nearing et al., 2021a) showing a strong correlation (*r*^2^ = 0.99, *p* < 0.011) with stimulus intensity (Libbus et al., 2016; Nearing et al., 2021a). The VNS-induced reduction TWA magnitude from baseline to 1 and 3 years is illustrated (Figure 5 right panels; Nearing et al., 2021a). This observation may help to explain in part the neutral results of the NECTAR-HF study, which employed a VNS stimulus intensity of 1.2 mA, nearly half of the stimulus intensity used in ANTHEM-HF Pilot study (Table 2). Such a 20-μV change in TWA has previously been found to be associated with decreases of > 55% in risk for cardiac mortality and > 58% for sudden cardiac death (Leino et al., 2011). At 36 months, TWA had returned to normal levels (<47 μV) in most patients (Verrier et al., 2011; Nearing et al., 2021a). VNS also decreased R-wave heterogeneity (RWH) and T-wave heterogeneity (TWH) (Figure 6; Nearing et al., 2021a). Concurrent with decreasing TWA, HRT, RWH, and TWH, VNS reduced from 11 to 3 (73%) the number of patients with non-sustained ventricular tachycardia (NSVT) > 3 beats across the 3-year period of observation (Figures 5, 6; Nearing et al., 2021a). It is important to emphasize that the analysis was based on more than 2,700 h of AECGs recorded over a 3-year period, including serial determinations of multiple autonomic measures, LVEF, cardiac electrophysiological indices, and arrhythmia incidence.

Regarding underlying electrophysiological mechanisms, TWA has been shown to reflect temporal and spatial heterogeneity of repolarization (Verrier et al., 2009, 2011; Weiss et al., 2011; Verrier and Ikeda, 2013). TWH, a well-documented precursor of TWA, has likewise been extensively studied in preclinical models and shown to correlate with susceptibility to ventricular fibrillation under diverse conditions of sympathetic nerve stimulation, vagus nerve activation, myocardial ischemia, and pharmacologic interventions (Verrier and Huikuri, 2017). RWH and TWH based on resting 12-lead ECGs were found to predict cardiac mortality and sudden cardiac death in the 5600-subject Health Survey 2000 (Kentta et al., 2016). Furthermore, RWH and TWH predicted ventricular fibrillation and appropriate implantable cardioverter defibrillator discharge in patients with ischemic and non-ischemic cardiomyopathy undergoing electrophysiological study (Tan et al., 2017).

## Effects of Chronic Vagus Nerve Stimulation on Left Ventricular Ejection Fraction and New York Heart Association Class

**FIGURE 7:**
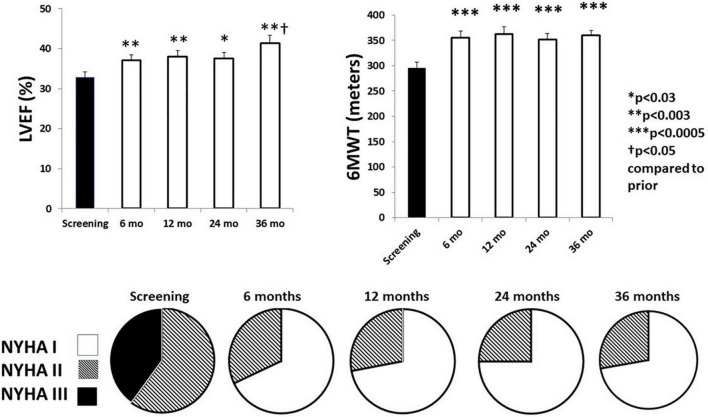
Effects of chronic vagus nerve stimulation (VNS) therapy on indices of cardiac performance. Significant increases in left ventricular ejection fraction (LVEF) **(upper left panel)** were observed across the 3-year observation period. A beneficial effect was also observed during the 6-min walk test (6MWT) **(upper right panel)**. All patients exhibited an improvement in New York Heart Association (NYHA) class status **(lower panel)**. By 6 months, NYHA class III patients (filled) were all reclassified to NYHA class I (open) or class II (striped). Reproduced with permission from Elsevier from Nearing et al. (2021a).

In association with the favorable effects of VNS on autonomic function and cardiac electrical stability, there was a sustained beneficial effect on LVEF, 6-min walk test, and NYHA class (Figure 7; Nearing et al., 2021a). It is notable that NYHA class was improved in 96% of patients. Potential mechanisms for VNS-mediated improvement in LVEF include, as discussed earlier, multiple sites of action, particularly protection of cardiac myocytes *via* a decrease in oxidative stress, apoptosis, and inflammatory response, as well as blunting the cardiotoxic effects of excessive levels of catecholamines through muscarinic-receptor mediated accentuated antagonism (Kolman et al., 1975; Levy and Blattberg, 1976; Li and Olshansky, 2011).

## Therapeutic Implications, Research Gaps, and Potential for Clinical Implementation

In this review, we discussed three main motivating factors that have prompted intense exploration of the potential use of VNS in addressing the increased morbidity and mortality associated with advanced heart failure. The first consideration is that despite the use of guideline-directed medical therapy, patients ultimately develop worsening of heart failure with progression to premature death. The second aspect is that heart failure progression has been linked in part to increased autonomic dysfunction. The third factor is that a sizeable body of preclinical studies indicates multimodal cardioprotective effects of VNS on autonomic function, cardiac mechanical performance, and reduced risk for malignant ventricular arrhythmias. Early clinical studies using VNS appeared promising; however, the larger INOVATE-HF and NECTAR-HF were neutral with respect to the primary clinical endpoints. The only sizeable study with promising results has been the ANTHEM-HF Pilot study (Premchand et al., 2014, 2016; Libbus et al., 2016; Nearing et al., 2021a; Sharma et al., 2021). The main apparent distinctive feature of ANTHEM-HF was use of the neural fulcrum to ensure a titration period in which autonomic engagement could be confirmed in each patient. Thus, it is useful to adhere to the historical rationale used in pharmacologic therapy, namely, “to administer the right drug, to the right patient, by the right route, at the right time, in the right amount, for the right duration” (Hutchinson et al., 1990).

Drawing on the substantial body of information provided by prior preclinical and clinical studies, the Autonomic Regulation Therapy to Enhance Myocardial Function and Reduce Progression of Heart Failure with Reduced Ejection Fraction (ANTHEM-HFrEF) Pivotal study (NCT03425422) (Houston, TX, United States) has been undertaken (Konstam et al., 2019). It is a large study, with a projected enrollment of 500–1,000 patients, and employs an adaptive, open-label, randomized controlled design. The rationale for anticipating a reasonably favorable outcome is that this study builds on knowledge gained from prior investigations to optimize VNS dosing, stimulation characteristics, and choice of appropriate patients, together with an innovative adaptive design for guiding sample size, as allowed by the new United States Food and Drug Administration breakthrough device program. The projected completion date is December 2024.

Thus, the clinical utility of neurostimulation in general and VNS in particular remains to be determined. Lest investigators and clinicians become impatient with the viability of neuromodulation approaches to the management of heart failure, as pointed out by Byku and Mann (2016), “it is instructive to recall that cardiac resynchronization therapy, which is now a class I indication in heart failure patients, took over two decades to evolve from a concept in animal models to widespread clinical application.” The ANTHEM-HFrEF Pivotal study could accelerate this timescale, given that the first-in-man study of VNS for heart failure was published by Schwartz et al. (2008).

## Author Contributions

All authors listed have made a substantial, direct, and intellectual contribution to the work, and approved it for publication.

## Conflict of Interest

RV and BN receive research funding from LivaNova USA. BK and IL are employees of LivaNova USA.

## Publisher’s Note

All claims expressed in this article are solely those of the authors and do not necessarily represent those of their affiliated organizations, or those of the publisher, the editors and the reviewers. Any product that may be evaluated in this article, or claim that may be made by its manufacturer, is not guaranteed or endorsed by the publisher.

## Abbreviations

ANTHEM-HF: Autonomic Regulation Therapy to Enhance Myocardial Function and Reduce Progression of Heart Failure Pilot Study (Houston,TX, United States)
ANTHEM-HFrEF: Autonomic Regulation Therapy to Enhance Myocardial Function and Reduce Progression of Heart Failure with Reduced Ejection Fraction Pivotal Study (NCT03425422) (Houston,TX, United States)
CardioFit: VNS device used in CardioFit and INOVATE-HF studies (BioControl Medical Ltd., Yehud, Israel)
HFrEF: heart failure with reduced ejection fraction
HRT: heart rate turbulence
HRV: heart rate variability
INOVATE-HF: INcrease Of VAgal TonE in Heart Failure (BioControl Medical Ltd., Yehud, Israel)
LVEF: left ventricular ejection fraction
LVESV: left ventricular end systolic volume
NECTAR-HF: Neural Cardiac Therapy for Heart Failure Study (Boston Scientific, Marlborough, MA, United States)
NSVT: non-sustained ventricular tachycardia
NYHA Class: New York Heart Association Class
rMSSD: heart rate variability measured as the square root of the mean of the squares of the successive differences between adjacent beats
RWH: R-wave heterogeneity
TWA: T-wave alternans
TWH: T-wave heterogeneity
VNS: vagus nerve stimulation.
